# Validation by collaborative trial of a method for the determination by GC–MS and LC–MS/MS of boar taint marker compounds in pork tissue

**DOI:** 10.1016/j.fochx.2020.100083

**Published:** 2020-03-04

**Authors:** Gerhard Buttinger, Thomas Wenzl

**Affiliations:** European Commission, Joint Research Centre (JRC), Retieseweg 111, B 2440 Geel, Belgium

**Keywords:** Boar taint, Skatole, Indole, Androstenone, Method validation by collaborative trial, Pork tissue, GC–MS, LC-MS/MS, Indole (PubChem CID: 798), Skatole (PubChem CID: 6736), 5α-Androst-16-en-3-one (PubChem CID: 6852393)

## Abstract

Meat from male pigs may develop an off-flavour, commonly known as boar taint. Castration of male piglets prevents the potential formation of off-flavour.

In the suggested method, three marker compounds for boar taint (skatole, androstenone and indole) are quantified in pork fat by isotope dilution gas chromatography mass spectrometry (GC–MS) or by isotope dilution liquid chromatography tandem mass spectrometry (LC–MS/MS).

This method was validated by collaborative trial according to ISO 5725-2:1994. The studied concentration ranges included sensorial thresholds. The repeatability relative standard deviation (RSD_r_) ranges from 3% to 10% and the reproducibility relative standard deviation (RSD_R_) from 10% to about 30%.

The method has proven to be robust and free from matrix interferences.

The method performance characteristics are compliant with requirements for official control methods in the area of food contaminants; therefore, the method is regarded as fit for its intended purpose.

## Highlights

• Simultaneous determination of indole, skatole, and androstenone in pork tissue.
• Sensorial thresholds are within working ranges of the method.
• Choice of sample measurement by GC–MS or LC–MSMS.
• Method validation by collaborative trial with participants from 10 countries.
• Method performance parameters are compliant with EU legislation on food contaminants.

## 1 Introduction

Rearing entire males instead of castrated male pigs for meat production has a number of advantages including lower production cost, leaner carcass, lower output of nitrogen in the environment, and reduction of suffering for the animal (Bonneau et al., 2000). However, meat from male pigs may develop on off-flavour, commonly known as boar taint. For that reason, male piglets are surgically castrated at young age to avoid the potential off-flavour formation. While castration may be legally performed without anaesthetics prior to seven days of age, available evidence suggests that castration at any age is painful (European Food Safety Authority, 2004).

Animal welfare concerns have triggered research into alternatives to surgical castration of male piglets with the long-term goal of abandoning it by 1 January 2018 (European Commission, 2010).

Skatole and androstenone (5α-androst-16-en-3-one) are the compounds considered primarily responsible for boar taint (Bonneau et al., 2000). The former might contribute to a larger extend to boar taint than the latter (Wauters et al., 2017). However, several factors such as sensorial sensitivity and form of presentation of tainted meat to the consumer influence the sensorial perception (Bekaert et al., 2013, Moerlein et al., 2013). Skatole is a tryptophan metabolite produced in the gut by microorganisms, while androstenone is a testicular steroid which is released into the bloodstream and accumulated in adipose tissue. Boar taint was occasionally also detected in barrows, gilts, and sows (Prusa et al., 2011). Concentrations between 0.50 μg/g and 1.00 μg/g for androstenone and between 0.20 μg/g and 0.25 μg/g for skatole are generally accepted as thresholds for quantitatively distinguishing differences between tainted and untainted pork back fat (Bonneau, 1998).

Various analytical methods of different sophistication can be used for the detection and quantification of boar taint compounds (Haugen, Brunius, & Zamaratskaia, 2012). Developments in this area date back to the late 1960s (Patterson, 1968). Since then, both gas chromatography and liquid chromatography were employed for the separation of different boar taint compounds, whereas mass spectromtric detection became also in this area state of the art (Haugen et al., 2012). While quite a number of mass spectrometry based and in-house validated confirmatory methods have been made available (Bekaert et al., 2012, Fischer et al., 2011, Fischer et al., 2012, Verplanken et al., 2016, Vandendriessche and Vanhaecke, 2012), rapid screening tests fit for sorting carcasses in slaughter houses are high in demand. Currently, sensory testing by the human nose is mostly applied but instrumental alternatives have been proposed as well (Lund, Minert, & Borggard, 2016). The European Commission funded the project “BoarCheck” to take stock of existing rapid methods, assess their usefulness and recommend those most relevant for boar taint detection at industrial level (European Commission, 2014).

Regrettably, none of the confirmatory methods has been validated by collaborative study so that it can be used as a point of reference for the development of rapid tests and the definition of sensory thresholds for consumer acceptance. Requirements for such a method were appropriate selectivity, a limit of quantification below the sensory thresholds of the three main compounds, ease of sample handling, and the flexibility to use different instrumental platforms. A Standard Operating Procedure (SOP) was developed based on an isotope dilution assay which gives the analyst the freedom to choose between a GC–MS or LC–MS/MS quantification step. This SOP was posted on the internet for commenting by interested parties, discussed at a workshop and consequently validated by collaborative study. The outcome of the study is reported here.

## 2 Materials and methods

### 2.1 Method description

The method quantifies the boar taint compounds indole, skatole and androstenone ((5α-androst-16-en-3-one) by an isotope dilution assay.

Fat is obtained from the back neck tissue via melting and separated by centrifugation from the remaining tissue material. An aliquot of the fat is spiked with isotope labelled analogues of the targeted compounds and purified by size exclusion chromatography (*SEC*) on Bio-beads S-X3® (Bio-Rad Laboratories N.V., Temse, Belgium), using a mixture of cyclohexane and ethyl acetate (1 + 1, v/v) as eluent. 100 μL of nonane or 1-octanol are added as a keeper to the collected *SEC* fraction, which is evaporated to about 100 μL. Finally, the sample is reconstituted in a solvent compatible with either GC–MS or LC–MS/MS for separation and quantification of the targeted compounds.

For GC–MS analysis the sample is injected splitless and separated on a capillary column coated with 5%-phenyl-methylpolysiloxane. The analytes are ionised by electron ionization (EI) at 70 eV, recorded in Single Ion Monitoring (SIM) mode and quantified using the isotopically labelled analogues.

For LC–MS/MS analysis, the separation is obtained on a sub-2-µm reversed phase C18 column. The analytes are ionised by atmospheric pressure chemical ionisation (APCI), detected in Selected Reaction Monitoring (SRM) mode and quantified using the isotopically labelled analogues.

A detailed Standard Operating Procedure is provided as Electronic Supplement.

## 3 Design of the collaborative study

Nine laboratories using LC–MS/MS and 15 laboratories employing GC–MS expressed their interest in participating in the collaborative trial, a few of them participating with both techniques. These laboratories were invited to a workshop, which had the aim to explain the design of the study, discuss questions regarding method details, and to show the practical implementation of the different analysis steps in the laboratory.

The study layout followed ISO 5725-2:1994 (ISO, 1994). The participants received five different test materials as blind-duplicates, covering the working range of the method. Additionally, the laboratories received one standard solution to estimate bias caused by calibration. Upon request, laboratories were also supplied with empty *SEC* columns and filling material.

Participating laboratories were requested to document all observations made while doing the analyses, in particular deviations from the testing protocol.

### 3.1 Samples

Two samples were prepared from naturally incurred pork. The fat tissue was frozen and homogenized using a meat grinder. Three samples were prepared by spiking lard; it was molten at 40 °C and after spiking stirred over night with a magnetic stir bar. The lard was pipetted into screw cap vials and all test materials were stored at −20 °C until dispatch.

A calibration check solution in toluene was prepared gravimetrically to be used for gas chromatography and in methanol for liquid chromatography based methods.

The homogeneity of the material was assessed by taking randomly 10 units and analysing them in duplicate by means of the LC–MS/MS method described in the Electronic Supplement. The results were evaluated according to ISO 13528:2015 (ISO, 2015) and all samples were found to be homogenous. An overview of prepared samples is given in Table 1. The presented concentration values are the average contents determined during homogeneity testing.

**Table 1:** Overview of prepared samples.

	Indole [µg/kg]	Skatole [µg/kg]	Androstenone [µg/kg]
Sample 1 tissue^1^	103	272	1946
Sample 2 tissue^1^	121	221	701
Sample 3 lard^1^	105	104	4113
Sample 4 lard^1^	386	1150	1160
Sample 5 lard^1^	1145	362	335
Calibration check solution in toluene^2^	226.1	221.8	1749.3
Calibration check solution in methanol^2^	244.5	234.7	1904.7

1 Values from homogeneity assessment by LC–MS/MS.

2 Values from gravimetric preparation.

The stability of the samples was verified employing an isochronous testing scheme where three units of each sample were stored at −20 °C (recommended storage temperature) and −70 °C (reference temperature where no change was expected) at the beginning of the study. After the last results were received all units were analysed in duplicate under repeatability conditions. The two sets of results were compared for significant differences. The analysis results for samples stored at the two temperatures were not significantly different, indicating sample stability, for all samples, except indole in sample 5 (spiked lard) and skatole in sample 1 (pork tissue). The content of indole decreased at −20 °C by about 15%, whereas about 40% of skatole was lost. However, the losses cannot be explained by sample degradation because of the fact that all other samples stayed stable. The data reported by the participants did not indicate any instability for these two analyte/sample combinations. The median of the results reported for skatole in sample 1 agreed well (245 µg/kg) with the mean value determined in the sample stored at reference temperature (255 µg/kg). The same holds true for indole in sample 5, with a median value of 985 µg/kg reported by the participants and a mean value measured in samples stored at the reference temperature of 975 µg/kg. Consequently, the data received for these two analyte/sample combinations were kept in the data pool and precision parameters were determined.

### 3.2 Data analysis

Statistical analysis of the reported data was done with PROlab™, a software package for the evaluation of interlaboratory studies (QuoData GmbH, Dresden, DE).

The evaluation was performed in three steps. They comprised of the evaluation of systematic effects, the evaluation of whether data obtained by the two measurement techniques can be pooled, and finally the calculation of precision parameters.

## 4 Results and discussion

Fifteen laboratories reported results. The plotted data were searched for pathologies and, in combination with Mandel's *h* respectively Mandel's *k* tests as stipulated in ISO 5725-2:1994, several laboratories were identified as stragglers. They were contacted and requested to investigate potential reasons for their deviating results. Depending on the reply of the laboratories, it was decided either to exclude results for technical reasons from the data pool for a particular analyte in a particular group of samples (lard samples or pork tissue samples) or for all samples.

Four laboratories identified technical reasons for reporting outlying indole results, three for skatole and four for androstenone. Among the reasons were analyte carry-over, calibration problems, inappropriate storage of sample, and difficulties to implement the method correctly. Therefore, the concerned data points were excluded from further evaluation.

**Fig. 1:**
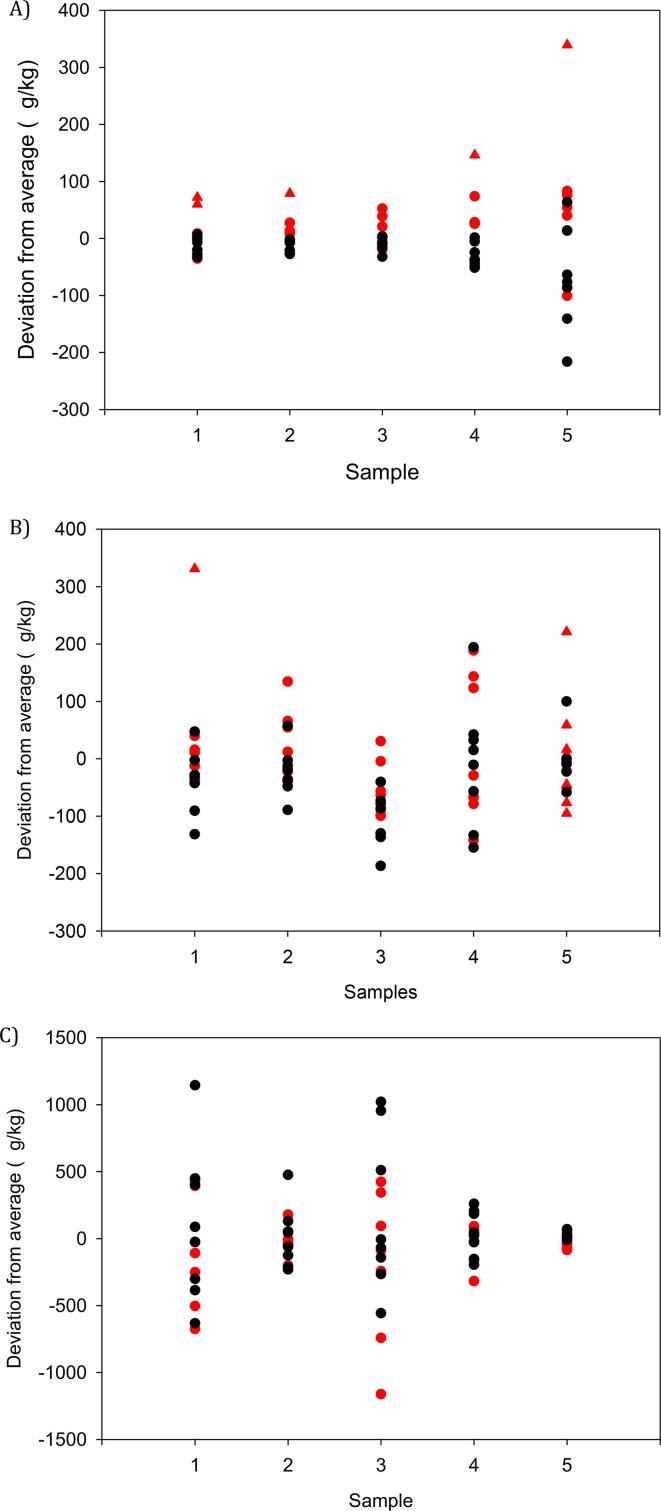
Compilation of means of valid blind duplicate analytical results reported by participants for A) indole, B) skatole, C) androstenone (LC–MS/MS represented by red symbols, GC–MS by black symbols, triangles represent outliers identified either by Cochran or Grubbs tests). (For interpretation of the references to colour in this figure legend, the reader is referred to the web version of this article.)

The means of technically valid results reported for blind duplicate measurements are depicted in Fig. 1; data have been centred for each sample on the arithmetic mean for better comparison purposes.

The equivalence of results obtained by LC–MS/MS and GC–MS was checked in order to find out whether pooling of results would be possible for the calculation of performance parameters. This was done by comparing the arithmetic mean values of the data obtained by the two techniques using the *t*-test (95% confidence level). Values for the mean and the standard deviation were calculated on the whole data pool without removing statistical outliers. Besides the agreement of the mean values, the equality of variances was evaluated by performing F-tests (95% confidence level). As no significant differences were found, the two measurement techniques were considered equivalent and the data were pooled for the estimation of the precision parameters. Precision parameters such as repeatability standard deviations, reproducibility standard deviations, repeatability limits and reproducibility limits were estimated according to ISO 5725-2:1994 after application of the outlier tests (Cochran and Grubbs) foreseen in the standard. An overview of the obtained results together with the respective Horwitz (HorRat) ratios for reproducibility is given in Table2. The latter parameter compares the actually obtained reproducibility standard deviations against a theoretical value derived from the Horwitz equation, which is a useful index of method performance with respect to precision (Horwitz & Albert, 2006). HorRat values <2 are considered to be fit-for-purpose. The HorRat ratios were for most analyte/sample combinations around 1, exempt for skatole in sample 3 and androstenone in sample 1, which were in both cases close to 2. The reason for these, compared to the other data, rather high values is not clear. However, they are still considered fit-for-purpose.

**Table 2:** Statistical evaluation of reported results reported.

		Indole					Skatole					Androstenone				
		Pork tissue samples		Spiked lard samples			Pork tissue samples		Spiked lard samples			Pork tissue samples		Spiked lard samples		
		Sample 1	Sample 2	Sample 3	Sample 4	Sample 5	Sample 1	Sample 2	Sample 3	Sample 4	Sample 5	Sample 1	Sample 2	Sample 3	Sample 4	Sample 5
No. of laboratories that submitted results		13	13	14	14	14	15	15	16	16	16	15	15	16	16	16
Median	µg/kg	106.00	113.00	78.25	372.50	985.70	245.75	190.10	116.10	1131.24	374.50	2338.50	806.19	3788.90	1137.00	315.77
Assigned value	µg/kg	103	121	105	386	1145	272	221	104	1150	362	1946	701	4113	1160	335
Reproducibility s.d.	µg/kg	16.55	17.52	24.03	40.93	99.52	48.43	57.29	46.26	117.86	48.85	532.23	129.82	579.77	157.54	45.68
Repeatability s.d.	µg/kg	5.03	5.24	8.85	12.70	47.64	13.79	13.32	9.41	44.57	12.40	193.01	51.31	136.67	50.00	13.19
Rel. reproducibility s.d.	%	16.07%	14.48%	27.30%	10.34%	9.51%	17.81%	25.92%	41.30%	10.43%	13.80%	27.66%	18.52%	14.38%	13.24%	16.43%
Rel. repeatability s.d.	%	4.88%	4.33%	10.05%	3.21%	4.55%	5.07%	6.03%	8.40%	3.94%	3.50%	10.03%	7.32%	3.39%	4.20%	4.75%
Limit of reproducibility, R (2.77 X sR)	µg/kg	45.84	48.53	66.56	113.37	275.68	134.15	158.68	128.14	326.47	135.32	1474.28	359.60	1605.96	436.39	126.52
Limit of repeatability, r (2.77 X sr)	µg/kg	13.92	14.50	24.51	35.18	131.97	38.20	36.90	26.06	123.45	34.35	534.65	142.13	378.59	138.50	36.55
HORRAT (R)		0.7	0.7	1.2	0.6	0.6	0.9	1.3	1.9	0.7	0.7	1.9	1.1	1.1	0.8	0.8
Absolute classical Horwitz s.d.	µg/kg	23.20	26.60	20.29	72.83	166.20	52.93	44.37	24.91	177.47	66.21	278.92	118.30	523.02	185.45	53.92
Relative classical Horwitz s.d.	%	22.52%	21.98%	23.06%	18.39%	15.89%	19.46%	20.08%	22.24%	15.71%	18.70%	14.50%	16.88%	12.97%	15.58%	19.40%
Type A outliers		0	0	0	0	0	0	0	0	0	0	0	0	0	0	0
Type B outliers		1	1	0	0	1	0	0	0	0	1	0	1	0	0	0
Type C outliers		1	0	0	1	0	1	0	1	0	0	1	0	0	0	1
No. of laboratories after elimination of outliers type A-C		11	12	14	13	13	14	15	15	16	15	14	14	16	16	15
No. of measurement values		26	26	28	28	27	30	30	32	32	31	30	30	32	32	31
No. of measurement values without outliers		22	24	28	26	25	28	30	30	32	29	28	28	32	32	29

Outlier types:

A: Single outlier – Grubbs test.

B: Differing laboratory mean – Grubbs test.

C: Excessive laboratory standard deviation - Cochran test.

Precision did not depend on the nature of the samples, as the numerical estimates were similar for pork tissue samples and for lard samples, indicating that melting and separating of the fat from the tissue did not have a major impact on analytical precision. However, reproducibility was on most occasions considerably larger than repeatability, which is an indicator that certain effects influenced variation among laboratories. Preparation of the calibration standards and instrument calibration must not be ignored in this respect and from the Mandel's *h* plots (Supplementary Fig. 1) it was evident that for almost all laboratories deviations from the overall mean values into a certain direction occurred, indicating bias.

**Fig. 2:**
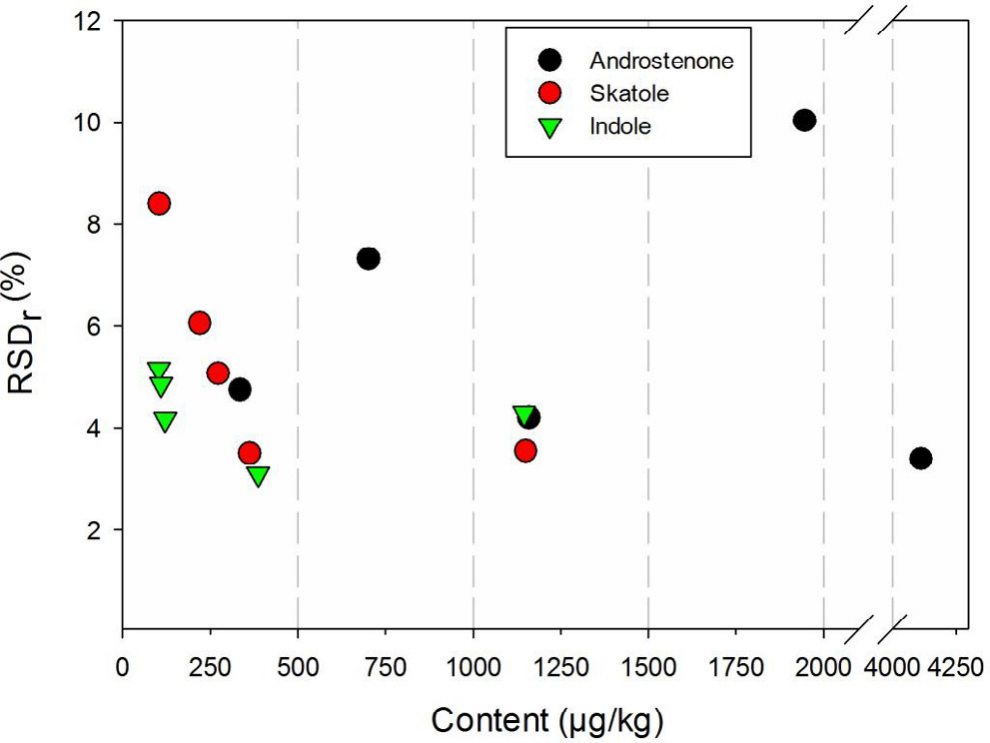
Dependence of repeatability relative standard deviation (RSD_r_) on analyte content.

**Fig. 3:**
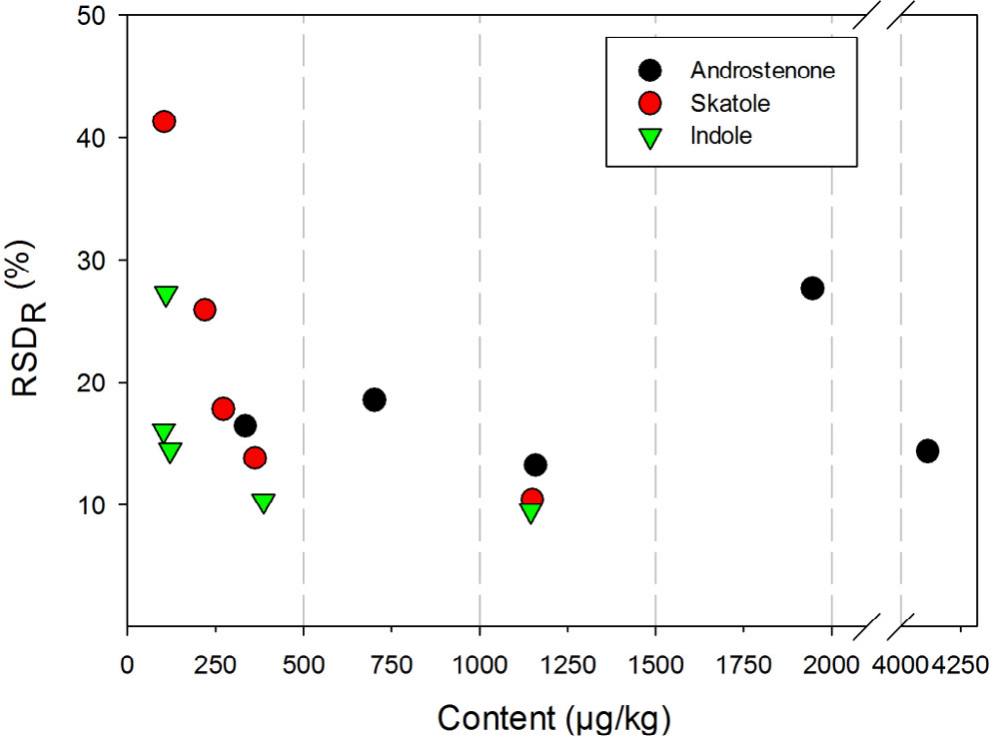
Dependence of reproducibility relative standard deviation (RSD_R_) on analyte content.

Repeatability relative standard deviations were similar for all three analytes as were the reproducibility relative standard deviations, albeit at higher levels (Fig. 2, Fig. 3). No pronounced analyte concentration dependency of the RSDs was observed, except for androstenone at the lowest tested concentration.

## 5 Conclusions

A method for the determination of the boar taint compound indole, skatole and androstenone was validated by collaborative trial. It fulfils the requirements for precision and is sensitive enough to determine the compounds at levels relevant for sensory perception. The validated range is 90–970 μg/kg for indole, 210–1150 μg/kg for skatole and 320–3850 μg/kg for androstenone in pork adipose tissue and lard. The analysis time, the sophistication of employed equipment, and the technical skills required for performing the analyses do not meet slaughterhouse requirements. However, the analytical method was intended to support arbitration. Additionally, analytical results obtained by this method may serve as references for the characterisation of rapid methods for the determination of boar taint in pork meat.

## Declaration of Competing Interest

The authors declare that they have no known competing financial interests or personal relationships that could have appeared to influence the work reported in this paper.
