# Supplementary material for: Validation by collaborative trial of a method for the determination by GC–MS and LC–MS/MS of boar taint marker compounds in pork tissue
Source: Food Chem X. 2020 Mar 4;6:100083. doi: 10.1016/j.fochx.2020.100083 (PMC7082526; doi:10.1016/j.fochx.2020.100083)
Supplement: Supplementary data 1 [file mmc1.docx]

SOP

Determination of boar taint compounds indole, skatole and androstenone in pork neck hypodermis.

# Scope

The three marker compounds for boar taint (skatole, androstenone and indole), are quantified in pork fat either by gas-chromatography with mass-spectrometry detection (GC-MS) or liquid chromatography with tandem mass spectrometry (LC-MS/MS).

# Principle

The fat is separated from the pork fat tissue sample via melting and separated via centrifugation from the remaining tissue material. Boar taint compounds in an aliquot of the fat , spiked with isotope labelled boar taint compounds, are purified by means of size exclusion chromatography (SEC), using a mixture of cyclohexane and ethyl acetate as eluent.

For GC or UHPLC analysis 100 μL of nonane or 1-octanol, respectively are added as a keeper to the collected SEC fraction. The SEC fraction is then evaporated to about 100 μL. Finally, the sample is reconstituted using an injection standard prior to measurement by GC-MS or LC-MS/MS.

For GC-MS analysis:

The injection is performed with a splitless injection port. The chromatographic separation is obtained on a capillary column with 5%-phenyl-methylpolysiloxane as stationary phase. The analytes are ionised by electron ionization (EI) at 70 eV. The target ions are recorded in Single Ion Monitoring (SIM) mode, and quantified by an isotope dilution method.

For UHPLC-MS/MS analysis:

The chromatographic separation is obtained on a sub-2-µm reversed phase C18 column. The analytes are ionised by atmospheric pressure chemical ionisation (APCI). The target boar taint compounds are detected in Selected Reaction Monitoring (SRM) mode and quantified by an isotope dilution method.

For HPLC-MS/MS analysis see Annex 1.

# Definitions

Laboratory sample: sample as prepared for sending to the laboratory and intended for inspection or testing (i.e. the sample or subsample(s) received by the laboratory).

Test sample: sample prepared from the laboratory sample and from which test portions will be taken.

Test portion: the quantity of material drawn from the test sample and on which the test or observation is actually carried out (i.e. for this study the test portion is of 4 g).

Final extract: solution containing the analytes; obtained after the last evaporation step and reconstitution of the extract.

Procedural blank: a blank sample made up of all reagents foreseen for the preparation of a test portion and processed in all respects as a test portion. This kind of blank, tests the purity of the reagents but also other possible sources of contamination, like the glassware and the analytical instrument (for this study 4 g of vegetable oil (e.g. palm oil), 2 g of sodium sulphate (4.13)).

# Reagents

## General

Use only reagents of recognized analytical quality/standard, unless otherwise specified. Commercially available solutions with equivalent properties to the reagents listed may be used.

Standard solutions are prepared gravimetrically. For the preparation of solutions of native or labelled boar taint compounds, a micro-balance (6.4) and an analytical balance (6.5) are used. All quantities are expressed as mass fractions (weight/weight). If necessary, the quantities expressed as mass concentration (weight/volume) could be obtained applying the density equation.

Density of toluene at 20°C is 0.8669 g/ml. All solutions and substances are used at room temperature.

**WARNING 1** — Indole is considered a potential carcinogen and just like its derivatives it is irritating to eyes, respiratory system and skin.

People using these instructions should be familiar with normal laboratory practice. It is the responsibility of the user of these instructions to apply safety and health practices which are in agreement with the local requirements.

## Helium purified compressed gas

(purity equivalent to 99.995% or better)

## Nitrogen purified compressed gas

(purity equivalent to 99.995% or better)

## Water

Type 1 (ASTM D1193)

## *n*-Hexane

## Methanol

HPLC grade or better

## Cyclohexane

HPLC grade or better

## 1-Octanol

HPLC grade or better

## Toluene

GC grade or better

## Ethyl acetate

HPLC grade or better

## Formic acid

Purity 98 % or better

## Nonane

Purity 99 % or better

## Sodium sulphate, anhydrous

p. A.

## Size exclusion chromatography (SEC) eluent

Mix 1 part per volume of cyclohexane (4.7) with 1 part per volume of ethyl acetate (4.10).

## LC eluent A: 0.1 % (v:v) aqueous formic acid

Mix 1 part per volume of formic acid (4.11) with 1000 parts per volume of water (4.4).

## LC eluent B: 0.1 % (v:v) methanolic formic acid

Mix 1 part per volume of formic acid (4.11) with 1000 parts per volume of methanol (4.6).

## Reference material for quality control

In descending order of preference, a certified reference material, or during a proficiency test round tested, or a self-prepared test material may be applied for this purpose.

This material will be analysed with every sample batch and used to control the method performances along time.

## Native reference standards

Commercially available neat material or solutions of boar taint compounds.

The list of native substances analysed with this method is reported in Table 1.

Commercially available, preferably certified, standard solutions are preferred due to the higher level of safety in handling.

Table 1 Names and structure of boar taint compounds and injection standard

| Name | CAS # | Structure |
| --- | --- | --- |
| Androstenone (5α-Androst-16-en-3-one) | 18339-16-7 |  |
| Indole (1H-Benzo[b]pyrrole) | 120-72-9 |  |
| Skatole (3-Methylindole) | 83-34-1 |  |
| 5-Chloroindole (5-Cl-indole, used as injection standard) | 17422-32-1 |  |

## Labelled reference standards

In form of neat material or commercially available solutions.

The labelled standards, applied for the quantification of the target boar taint compounds included in the scope of this instruction are listed in Table 2.

***Remark***

The labelled standard androstenone-d4 has 4 deuterium atoms in alpha positions to the carbonyl group in ring A. It is well known that deuterium can be lost from such a position via the keto/enol tautomerism (it is indeed the way of production of the labelled compound). Therefore, a back exchange in methanolic solutions is possible. It is recommended to keep methanolic solutions containing androstenone-d4 at -20 °C, although no change in isotope ratio could be detected in a methanolic solution after 1 year at 4 °C.

Table 2 List of labelled boar taint compounds

| Labelled standard | CAS # | Structure |
| --- | --- | --- |
| Androstenone-d4  (5α-Androst-16-en-3-one-2,2,4,4-d4) |  |  |
| Indole-d7 | 73509-20-3 |  |
| Skatole-d3  (3-Methyl-d_3_-indole) | 111399-60-1 |  |

## 5-Chloroindole

Neat material or commercially available solutions.

5-Chloroindole (Table 1) is used as injection standard and added to the sample extract prior to GC-MS or LC-MS/MS analysis.

# Standard preparation

## Injection standard stock solution

Prepare gravimetrically a 20 mL solution of 5-chloroindole (4.20) in toluene (4.9) with a concentration of approximately 5 mg/mL (i.e. 100 mg neat substance). The conversion of units to mg/mL is done via the density equation. For toluene, a density value of 0.8669 g/mL is applied at 20 °C.

## Methanolic injection standard stock solution

Prepare gravimetrically a 20 mL solution of 5-chloroindole in methanol (4.6) with a concentration of approximately 50 µg/mL based on the injection standard stock solution (5.1) (i.e. 200 µl of 5.1). The conversion of units to µg/mL is done via the density equation. For methanol, a density value of 0.7918 g/mL is applied at 20 °C.

## Methanolic injection standard solution

Prepare gravimetrically a solution of 5-chloroindole in methanol (4.6) with a concentration of approximately 250 ng/mL based on the methanolic injection standard stock solution (5.2). The conversion of units to µg/mL is done via the density equation. For methanol, a density value of 0.7918 g/mL is applied at 20 °C.

## Toluene injection standard stock solution

Prepare gravimetrically a 20 mL solution of 5-chloroindole in toluene (4.9) with a concentration of approximately 50 µg/mL based on the injection standard stock solution (5.1) (i.e. 200 µl of 5.1). The conversion of units to µg/mL is done via the density equation. For toluene, a density value of 0.8669 g/mL is applied at 20 °C.

## Toluene injection standard solution

Prepare gravimetrically a solution of 5-chloroindole in toluene (4.9) with a concentration of approximately 250 ng/mL based on the toluene injection standard stock solution (5.4). The conversion of units to ng/mL is done via the density equation. For toluene, a density value of 0.8669 g/mL is applied at 20 °C.

## Native reference standards single-substance stock solutions

Prepare gravimetrically from the native reference standards (4.18) skatole and indole, individual solutions in toluene (4.9) with a concentration of approximately 5 mg/mL. Prepare gravimetrically from the androstenone native reference standard a solution in toluene (4.9) with a concentration of approximately 1.5 mg/mL.

Single-substance stock solutions prepared by weighing a limited amount of native reference standard (below 20 mg) should be prepared by accurate weighing. The amount of reference standard should be determined in a glass weighing cylinder (6.1) using the microbalance (6.4). For amounts above 50 mg an analytical balance can be used. After determination of the amount of standard present in the weighing cylinder, cylinder plus standard are transferred into an amber volumetric flask (6.2) and its weight is determined using an analytical balance (6.5). Subsequently, the appropriate amount of toluene (4.9) is added and weighted with an analytical balance (6.5). For toluene a density value of 0.8669 g/mL is applied at 20 °C.

To dissolve the substances, each solution shall be sonicated for a couple of minutes.

Once the solutions are homogeneous, they are transferred into amber glass vials (6.6) and stored in the dark and at a temperature below 10 °C.

## Methanolic indole and skatole stock solution (25 µg/mL)

Prepare, from the single-substance stock solutions of indole and skatole (5.6), gravimetrically a solution in methanol (4.6) with a concentration of approximately 25 μg/mL. For this purpose, both the single standard stock solutions (5.6) and methanol (4.6) are weighed with an analytical balance (6.5). The conversion of units to µg/mL is done via the density equation. For methanol, a density value of 0.7918 g/mL is applied at 20 °C.

The solution is homogenised by shaking and subsequently transferred into an amber glass vial (6.6) and stored in the dark and at a temperature below 10 °C.

## Methanolic indole and skatole stock solution (500 ng/mL)

Prepare, from the mixed methanolic indole and skatole stock solution (5.7), gravimetrically a solution in methanol (4.6) with a concentration of approximately 500 ng/mL. For this purpose, both the mixed methanolic stock solution (5.7) and methanol (4.6) are weighed with an analytical balance (6.5). For methanol, a density value of 0.7918 g/mL is applied at 20 °C.

The solution is homogenised by shaking and subsequently transferred into an amber glass vial (6.6) and stored in the dark and at a temperature below 10 °C.

## Methanolic androstenone solution (25 µg/mL)

Prepare, from the single-substance stock solution of androstenone (5.6), gravimetrically a solution in methanol (4.6) with a concentration of approximately 25 μg/mL. For this purpose, both the single standard stock solution (5.6) and methanol (4.6) are weighed with an analytical balance (6.5). For methanol, a density value of 0.7918 g/mL is applied at 20 °C.

The solution is homogenised by shaking and subsequently transferred into an amber glass vial (6.6) and stored in the dark and at a temperature below 10 °C.

## Labelled reference standards single-substance stock solutions

Prepare gravimetrically from the labelled reference standards (4.19) skatole-d3, indole-d7, and androstenone-d4, individual solutions in toluene (4.9) with a concentration of approximately 100 µg/mL.

Single-substance stock solutions prepared by weighing a limited amount of labelled reference standard (below 20 mg) should be prepared by substitution weighing. For amounts above 50 mg an analytical balance can be used. The amount of labelled standard should be determined in a glass weighing cylinder (6.1) using the microbalance (6.4). After determination of the amount of standard present in the weighing cylinder, cylinder plus standard are transferred into an amber volumetric flask (6.2) and its weight is determined using an analytical balance (6.5). Subsequently, the appropriate amount of toluene (4.9) is added and weighted with an analytical balance (6.5). The conversion of units to µg/mL is done via the density equation. For toluene, a density value of 0.8669 g/mL is applied at 20 °C.

To dissolve the substances, each solution shall be sonicated for a couple of minutes.

Once the solutions are homogeneous, they are transferred into amber glass vials (6.6) and stored in the dark and at a temperature below 10 °C.

## Labelled standards process solution

Prepare, with the individual solutions of skatole-d3, indole-d7, and androstenone-d4 (5.10) a solution in toluene (4.9) with a concentration of 8 µg/mL androstenone-d4, 4 µg/mL skatole-d3, and 2 µg/mL indole-d7. For toluene a density value of 0.8669 g/mL is applied at 20 °C. For this purpose, both the labelled single standard stock solutions (5.10) and toluene (4.9) are weighed with an analytical balance (6.5).

This solution will be used for spiking the test portion (7.2). The solution is homogenised by shaking and subsequently transferred into an amber glass vial (6.6) and stored in the dark and at a temperature below 10 °C.

## Methanolic labelled standards calibration solution

Prepare, with the individual solutions of skatole-d3, indole-d7and androstenone-D_4_ (5.10) and of 5‑Cl-indole (5.2) a solution in methanol (4.6) with a concentration of approximately 4 µg/mL androstenone-d4, 2 µg/mL skatole-d3, and 1 µg/mL indole-d7 and 2 µg/mL 5-Cl-indole. For this purpose, both the labelled single standard stock solution (5.10), (5.2) and methanol (4.6) are weighed with an analytical balance (6.5). For methanol, a density value of 0.7918 g/mL is applied at 20 °C.

This solution will be used for preparation of the methanolic calibration stock solutions (5.13). The solution is homogenised by shaking and subsequently transferred into an amber glass vial (6.6) and stored in the dark and at a temperature below 10 °C.

## Methanolic calibration stock solutions

Prepare gravimetrically the calibrants presented in Table 3 in methanol (4.6) using the labelled standards calibration solution (5.12), the methanolic indole and skatole solutions (5.7 or 5.8), the methanolic androstenone solutions (5.9).

Table 3. Nominal target concentrations in methanolic calibration stock solutions.

| Calibrant stock | indole D_7_ | skatole D_3_ | androstenone D_4_ | 5-Cl-indole | indole | skatole | androstenone |
| --- | --- | --- | --- | --- | --- | --- | --- |
|  | Nominal concentration in ng/mL | | | | | | |
| CAL 1 | 100 | 200 | 400 | 200 | 0 | 0 | 0 |
| CAL 2 |  |  |  |  | 50 | 50 | 100 |
| CAL 3 |  |  |  |  | 100 | 100 | 250 |
| CAL 4 |  |  |  |  | 150 | 150 | 500 |
| CAL 5 |  |  |  |  | 200 | 200 | 1 500 |
| CAL 6 |  |  |  |  | 400 | 400 | 2 500 |
| CAL 7 |  |  |  |  | 600 | 600 | 3 500 |
| CAL 8 |  |  |  |  | 900 | 900 | 4 500 |

Table 4. Volumetric preparation scheme to prepare 10 mL of the methanolic calibrant stock solutions

|  | Indole D_7_  skatole D_3_ androstenone D_4_ 5-Cl-indole | Indole skatole | androstenone | methanol |
| --- | --- | --- | --- | --- |
| CAL 1 | 1 000 µL  (5.12) | - | - | 9 000 µL |
| CAL 2 |  | 1 000 µL (5.8) | 50 µL (5.9) | 7950 µL |
| CAL 3 |  | 2 000 µL (5.8) | 100 µL (5.9) | 6 900 µL |
| CAL 4 |  | 60 µL (5.7) | 200 µL (5.9) | 8 740 µL |
| CAL 5 |  | 80 µL (5.7) | 600 µL (5.9) | 8 320 µL |
| CAL 6 |  | 160 µL (5.7) | 1 000 µL (5.9) | 7 840 µL |
| CAL 7 |  | 240 µL (5.7) | 1 400 µL (5.9) | 7 360 µL |
| CAL 8 |  | 360 µL (5.7) | 1 800 µL (5.9) | 6 840 µL |

## Toluene indole and skatole stock solution (25 µg/mL)

Prepare, from the single-substance stock solutions of indole and skatole (5.6), gravimetrically a solution in toluene (4.9) with a concentration of approximately 25 μg/mL. For this purpose, both the single standard stock solutions (5.6) and toluene (4.9) are weighed with an analytical balance (6.5). The conversion of units to µg/mL is done via the density equation. For toluene, a density value of 0.8669 g/mL is applied at 20 °C.

The solution is homogenised by shaking and subsequently transferred into an amber glass vial (6.6) and stored in the dark and at a temperature below 10 °C.

## Toluene indole and skatole stock solution (500 ng/mL)

Prepare, from the mixed toluene indole and skatole stock solution (5.14), gravimetrically a solution toluene (4.9) with a concentration of approximately 500 ng/mL. For this purpose, both the mixed toluene stock solution (5.14) and toluene (4.9) are weighed with an analytical balance (6.5). For toluene, a density value of 0.8669 g/mL is applied at 20 °C.

The solution is homogenised by shaking and subsequently transferred into an amber glass vial (6.6) and stored in the dark and at a temperature below 10 °C.

## Toluene androstenone solution (25 µg/mL)

Prepare, from the single-substance stock solution of androstenone (5.6), gravimetrically a solution in toluene (4.9) with a concentration of approximately 25 μg/mL. For this purpose, both the single standard stock solution (5.6) and toluene (4.9) are weighed with an analytical balance (6.5). For toluene, a density value of 0.8669 g/mL is applied at 20 °C.

The solution is homogenised by shaking and subsequently transferred into an amber glass vial (6.6) and stored in the dark and at a temperature below 10 °C.

## Toluene labelled standards calibration solution

Prepare, with the individual solutions of skatole-d3, indole-d7 and androstenone-d4 (5.10) and of 5‑Cl-indole (5.4) a solution in toluene (4.9) with a concentration of approximately 4 µg/mL androstenone-d4, 2 µg/mL skatole-d3, and 1 µg/mL indole-d7 and 2 µg/mL 5-Cl-indole. For this purpose, both the labelled single standard stock solution (5.10), (5.4) and toluene (4.9) are weighed with an analytical balance (6.5). For toluene, a density value of 0.8669 g/mL is applied at 20 °C.

This solution will be used for preparation of the toluene calibration stock solutions (5.18). The solution is homogenised by shaking and subsequently transferred into an amber glass vial (6.6) and stored in the dark and at a temperature below 10 °C.

## Toluene calibration stock solutions

Prepare gravimetrically the calibrants presented in Table 6 in toluene (4.9) using the labelled standards calibration solution (5.17), the toluene indole and skatole solutions (5.14or 5.15), the toluene androstenone solutions (5.16).

Table 5. Nominal target concentrations in toluene calibration stock solutions.

| Calibrant stock | indole D_7_ | skatole D_3_ | androstenone D_4_ | 5-Cl-indole | indole | skatole | androstenone |
| --- | --- | --- | --- | --- | --- | --- | --- |
|  | Nominal concentration in ng/mL | | | | | | |
| CAL 1 | 100 | 200 | 400 | 200 | 0 | 0 | 0 |
| CAL 2 |  |  |  |  | 50 | 50 | 100 |
| CAL 3 |  |  |  |  | 100 | 100 | 250 |
| CAL 4 |  |  |  |  | 150 | 150 | 500 |
| CAL 5 |  |  |  |  | 200 | 200 | 1 500 |
| CAL 6 |  |  |  |  | 400 | 400 | 2 500 |
| CAL 7 |  |  |  |  | 600 | 600 | 3 500 |
| CAL 8 |  |  |  |  | 900 | 900 | 4 500 |

Table 6. Volumetric preparation scheme to prepare 10 mL of the toluene calibrant stock solutions

|  | Indole D_7_  skatole D_3_ androstenone D_4_ 5-Cl-indole | Indole skatole | androstenone | toluene |
| --- | --- | --- | --- | --- |
| CAL 1 | 1 000 µL  (5.17) | - | - | 9 000 µL |
| CAL 2 |  | 1 000 µL (5.15) | 50 µL (5.16) | 7950 µL |
| CAL 3 |  | 2 000 µL (5.15) | 100 µL (5.16) | 6 900 µL |
| CAL 4 |  | 60 µL (5.14) | 200 µL (5.16) | 8 740 µL |
| CAL 5 |  | 80 µL (5.14) | 600 µL (5.16) | 8 320 µL |
| CAL 6 |  | 160 µL (5.14) | 1 000 µL (5.16) | 7 840 µL |
| CAL 7 |  | 240 µL (5.14) | 1 400 µL (5.16) | 7 360 µL |
| CAL 8 |  | 360 µL (5.14) | 1 800 µL (5.16) | 6 840 µL |

# Apparatus

**WARNING 3** — All glassware must be meticulously cleaned (except disposable glassware). The glassware is first thoroughly washed with laboratory detergent and hot water. All glassware is rinsed before use with *n*-hexane (4.5).

## Glass weighing cylinders,

approximately 1 mL volume

## Amber glass volumetric flasks,

of various volumes (5 mL to 100 mL)

## Reaction glass tubes (16 x 100 mm, Pyrex)

with PTFE layered screw caps

## Micro-balance,

with a readability of 0.000 001 g

## Analytical balance,

with a readability of at least 0.000 1 g

## Amber crimp cap glass vials

## Laboratory balance,

with a readability of 0.01 g

## (Ceramic) knife or scalpel,

## Heating device,

with the capability to heat the reaction tubes (6.3) to 90 °C ± 3 °C for at least 60 min

## Centrifuge

## Glass Pasteur capillary pipettes,

230 mm length

## PTFE^®^ membrane filter,

Ø 13 mm and 0.45 μm pore size

## Size Exclusion Chromatography (SEC) apparatus,

comprising the following

### Liquid pump,

suitable for a flow rate of 1.0 mL/min

### Sample carousel,

for vials of 2 mL capacity (optional)

### Sample collection vials,

20 mL capacity

### Injection system,

capable of injecting 750 μL.

### SEC column,

with the following characteristics: Bio-beads S-X3^®^ in 10 x 450 mm glass column, preconditioned in cyclohexane:ethly acetate = 1:1

Alternative columns may be applied provided that equality of performance is demonstrated.

The SEC column shall be kept following the supplier specifications.

### Solvent collection bottles

### Instrument control and data processing system,

e.g. computer based

### Autosampler vials for SEC apparatus

## Sample concentration apparatus:

sample concentrator, comprising a block heater capable of evaporating small volume samples at controlled temperature and under gas stream (e.g. Techne^®^ sample concentrator) (4.3)

## Microliter syringe(s) or calibrated microliter pipette(s),

with 25 μL to 500 μL capacity

## Gas-chromatography – mass spectrometry (GC-MS) apparatus,^[[1]](#footnote-1)^

comprising the following

### Injection system,

split/splitless injector, suitable for temperatures up to 400 °C.

### GC oven,

suitable for temperatures up to 350 °C and capable of temperature programming

### Sample tray

### Amber sample vials for the sample tray (6.16.3),

with a capacity of about 1.5 ml

### GC capillary column,

DB-5MS (5%-Phenyl-methylpolysiloxane), 30 m, 0.25 mm i.d., 0.25 μm film thickness. A capillary column with these characteristics shall ensure an acceptable resolution.

### An interface with the mass spectrometer,

with a temperature control device, suitable for temperatures up to 350ºC (see 9.1.4)

### Mass spectrometer

with the following characteristics:

- Electron Ionisation source with inert inner surface

- Ionisation energy of 70 eV

- Mass resolution: at least 1 atom mass unit (amu)

- Temperature control devices for the ion source (up to 300 °C), the quadrupole (up to 200 °C), the GC-MS interface (up to a 350 °C)

- Tuning stability at least of 48 h (allowing for the analysis of a sequence of samples or standards)

- Response linearity range of at least two orders of magnitude

### Computer based instrument control system,

capable of programming different acquisition modes in different time intervals.

### Data processing system,

computer based

## Liquid-chromatography – mass spectrometry (LC-MS/MS) apparatus^[[2]](#footnote-2)^,

comprising the following

### Liquid pump,

suitable for a flow rate of 0.3 mL/min and a back pressure of up to 1000 bar

### Sample carousel,

for vials of 2 mL capacity

### Injection system,

capable of injecting 10 μL.

### C18 column,

Macherey-Nagel, Nucleodur C18 Pyramid 1.8 μm 100 x 2.0 mm or equivalent

The column shall be kept following the supplier specifications. To extend column life it shall be protected with a 0.2 µm inline filter and if possible with a guard column.

### Solvent collection bottles

### Instrument control and data processing system,

e.g. computer based

### Autosampler vials for sample carousel

### Triple quadrupole mass spectrometer

with the following characteristics:

- atmospheric pressure chemical ionisation (APCI)

- mass resolution: at least 1 amu

# Procedure

For each batch of samples, a procedural blank shall be run in parallel, to assess interferences deriving from the applied reagents and apparatus. A reference material (quality control sample) shall be also included in the batch, for checking the method performances along time.

## Sample treatment

As a general precaution, all of the sample material received by the laboratory shall be used for obtaining a representative and homogeneous laboratory sample without introducing secondary contamination. This is achieved by blending the frozen sample or mincing.

**Remark**

**If the sample received contains not only subcutaneous fat tissue (hypodermis) but also skin, muscle or visceral fat (adipose tissue) these tissues have to be removed before homogenization.**

**Only the hypodermis is used for analysis!**

## Test portion preparation and fat extraction by melting

To obtain the test portion weigh, 2 g of sodium sulphate (4.13) into a reaction tube (6.3), thereafter weigh 4 g ± 0.1 g of the homogenised test sample into the reaction tube with an analytical balance (6.5).

Close the reaction tube and heat it for 1 h to 90 °C.

Centrifuge the tube at 40°C for 5 min at 3220 g.

Transfer 1 g ± 0.01 g (W_sample_) of the fat into a glass vial using a glass Pasteur pipette (6.11) and an analytical balance (6.5)

Add 100 μL of labelled process solution (5.11) for the quantification of the analytes by isotope dilution, add and dilute with 5 mL SEC eluent (4.14).

## SEC clean-up

Around 1.5 ml of test sample, prepared as described in 7.2, are filtered using a 0.5 µm syringe filter (6.12) into the SEC autosampler vial (6.13.8).

The SEC takes place under the following conditions:

Injection volume: 750 μL (if necessary adaptable to a maximum of 200 mg fat loaded on column)

Flow rate: 1 mL/min

Collected fraction: 25.0 min to 37.0 min (approximately 12 mL)

Eluent: 1:1 (v:v) cyclohexane: ethyl acetate (4.14)

## Preparation of the sample for the LC-MS/MS analysis

At the end of the SEC cleanup, 100 μL 1-octanol (4.8) are added to the collected fraction and the fraction is thereafter evaporated under a gentle stream of nitrogen down to 100 μL at 40 °C in the sample evaporation apparatus (6.14).

***Care has to be taken not to evaporate to dryness!***

Subsequently, the sample is reconstituted in 300 µL of methanolic injection standard solution (5.3) and thoroughly vortexed. The reconstituted sample is transferred into a 2 mL amber vial (6.17.7) for analysis.

## Preparation of the sample for the GC-MS analysis

At the end of the SEC cleanup, 100 μL of nonane (4.12) are added to the collected fraction and the fraction is thereafter evaporated under a gentle stream of nitrogen down to 100 μL at 40 °C, in the sample evaporation apparatus (6.14).

***Care has to be taken not to evaporate to dryness!***

Subsequently, the sample is reconstituted in 300 µL of toluene injection standard solution (5.5) and thoroughly vortexed. The reconstituted sample is transferred into the autosampler vial (6.16.4) for analysis.

# Sample analysis by LC-MS/MS

Chromatographic conditions have been optimized for the configuration of the UHPLC chromatograph (6.17) and the characteristics of the current column (6.17.4, column void volume of 187 µL)

## Instrumental conditions

### UHPLC conditions:

Injection volume: 5 μL

Column temperature: 28.4 °C

Flow rate: 0.3 mL/min

Gradient:

| Time | %LC eluent A (4.15) | Curve |
| --- | --- | --- |
| 0 | 39 | 6 |
| 3.58 | 3.6 | 6 |
| 4.80 | 3.6 | 6 |
| 4.81 | 39 | 6 |
| 6.50 | 39 | 6 |

Above conditions shall result in a representative chromatogram as shown in Figure 3. Indicative retention times are presented in Table 7.

Figure 3. Chromatogram of calibration standard CAL7 in 3:1 (v:v) methanol : 1-octanol (5µL injection volume)

Table 7. Indicative analyte retention times.

| **Compound** | **Retention time (min)** |
| --- | --- |
| Indole | 1.64 |
| Indole-d7 | 1.60 |
| Skatole | 2.12 |
| Skatole-d3 | 2.12 |
| 5-Chloroindole | 2.36 |
| Androstenone | 4.80 |
| Androstenone-d4 | 4.80 |

### Mass spectrometer acquisition parameters and peak identification

Analytes are ionised by positive mode atmospheric pressure chemical ionisation (APCI+) and specific fragment ions are detected after collision induced dissociation (CID) by selected reaction monitoring (SRM). Ionisation parameters are shown in Table 8. Detected SRM transitions and corresponding parameters are shown in Table 9. All measurements are performed with a span of 0.5 m/z, an Inter Channel Delay time of 5 ms and an Inter Scan Time of 5 ms. Dwell times shall be optimised to reach at least 12 points across the peak.

With reference to Commission Decision (EC) 2002/657 - Table 4 of the Annex, a tolerance of 10% to 50% in the value of the ratio is accepted, depending on the amount of the diagnostic ion in relation with the target ion (for this method qualifier ion transition Q_1_ and quantifier ion transition Q_2_ respectively).

A substance eluting from the chromatographic column is identified as one of the target analytes only:

- if the retention time of the unknown substance coincides with the retention time of the native compound ± 2 SD as found in the calibration solutions.
- if both quantifier and qualifier ion transitions are detected
- if for indole and skatole the Q_1_/Q_2_ peak area ratio values are within ± 2 SD as found in the calibration solutions. (during method development 7.9 ± 2.3 and 5.1 ± 1.4 respectively)
- if for 5-Cl-indole the peak area in the sample is within ± 2 SD as found in the calibration solutions.

Table 8. Ionisation parameters for Waters Quattro Premier

| Mode | Positive APCI |
| --- | --- |
| Corona discharge | 7.5 µA |
| Cone voltage | Depending on analyte (see below ) |
| Extractor | 5 V |
| RF Lens | 0 V |
| Source temperature | 120°C |
| Desolvation temperature | 600°C |
| Cone gas flow | 500 L/h |
| Desolvation gas flow | 100 L/h |
| Collision gas flow | 0.35 mL/min |
| Collision energy | Depending on analyte (see below) |
| Multiplier voltage | 650 V |

Table 9. SRM detection parameters for UHPLC-MS/MS analysis on Waters Quattro Premier instrument

| Time | SRM Transition | Dwell Time (s) | Cone Voltage | Collision Energy (eV) | Compound |
| --- | --- | --- | --- | --- | --- |
| 0.00-1.98 | 117.90 > 65.01 | 0.100 | 40.00 | 29.00 | Indole^‡^ |
|  | 117.90 > 90.97 | 0.100 | 40.00 | 20.00 | Indole^†^ |
|  | 123.97 > 95.88 | 0.100 | 40.00 | 20.00 | Indole-d7^†^ |
|  |  |  |  |  |  |
| 1.90-4.00 | 131.98 > 89.80 | 0.100 | 34.00 | 31.00 | Skatole^‡^ |
|  | 131.98 > 116.90 | 0.100 | 34.00 | 23.00 | Skatole^†^ |
|  | 134.98 > 116.90 | 0.100 | 34.00 | 23.00 | Skatole-d3^†^ |
|  | 151.95 > 116.95 | 0.100 | 30.00 | 18.00 | 5-Chloroindole^†^ |
|  |  |  |  |  |  |
| 4.00- 6.50 | 273.23 > 158.37 | 0.100 | 30.00 | 17.00 | Androstenone^‡^ |
|  | 273.23 > 255.12 | 0.100 | 30.00 | 14.00 | Androstenone^†^ |
|  | 277.23 > 259.12 | 0.100 | 30.00 | 14.00 | Androstenone-d4^†^ |
| ^†^: quantifier SRM transition | | | | | |
| ^‡^: qualifier SRM transition | | | | | |
|  | | | | | |

## Sample analysis

Before starting the sequence, two solvent blanks (methanol, 4.6) and the calibration standard solution CAL2 (5.13) have to be injected to verify the instrumental performances for this specific method. At the end of the sequence analysis of these solutions is repeated.

The chromatogram of the second solvent blank shall be checked for peaks which could indicate the need of cleaning the system.

The chromatogram of the CAL2 standard solution shall be examined to check the instrument's sensitivity and column performance, i.e. retention times, peak tailing, resolution between compounds.

In each sequence of analysis, after having checked the system, the calibrants, the procedural blank, the QC sample and the sample extracts shall be injected.

At the end of the sequence, results shall be checked to verify if any sample should be outside of the working range and, if necessary, the procedure described in 8.3.1 is applied.

## Data Analysis & Reporting

### Calibration

Calibration is performed by triplicate 5 µL injections of the calibration solutions, randomized along the sequence. All solutions shall be equilibrated to room temperature prior to injection.

The calibration curve is obtained by plotting the peak area ratio of the ion transition of the target compounds' quantifier and its corresponding labelled standard against the relative concentration of these compounds in the calibration solutions.

The calibration function is determined for each analyte by unweighted linear regression, and can be described by Equation 1.

$$\frac{A_{Q_{1}x}}{A_{Q_{1}IS_{x}}}=\alpha_{x}\frac{C_{x}}{C_{IS_{x}}}+\beta_{x}$$

Equation 1.

with *C* the mass fraction [ng/g] of *x*, the compound of interest, or its corresponding labelled standard *IS_x_*with *A* the quantifier ion transition peak area (Q_1_) relevant for *x*, respectively *IS_x_*
with *α_x_* and *ß_x_* respectively the slope and intercept of the calibration curve.

Prepare appropriate calibration curves in case the content of boar taint compounds in the sample should be outside the calibration range and within the linear range determined during the method validation. A new sample extraction shall be performed, adjusting the test portion weight so to obtain a final concentration of the boar taint compounds in the extract within the calibration range.

### Calculation

Equation 2 is used for the calculation of the boar taint compound mass fraction in the sample prepared as described in paragraphs 7.2 to 7.4.

$$[X]_{sample}=\frac{\left(\frac{\left(\frac{A_{Q_{1}x}}{A_{Q_{1}IS_{x}}}-\beta_{x}\right)}{\alpha_{x}}\right)S_{IS_{X}}}{W_{sample}}$$

Equation 2

with *[X]_sample_* the mass fraction [µg/kg] of *x,* the compound of interest, in the test portion.
with *A* the quantifier ion transition peak area (Q_1_) relevant for *x*, respectively *IS_x_* (the corresponding labelled standard for *x*).
with *α_x_* and *ß_x_* respectively the regression coefficients for *x* determined according to paragraph 8.3.1.
with *S_ISx_* the mass (µg) of labelled standard *IS_x_* spiked into the test portion (7.2).
with *W_sample_* the mass (kg) of test portion (7.2).

### Reporting

Analysis results will not be reported if the quality control criteria are not met. (8.1.2).

All results will be reported together with the corresponding expanded measurement uncertainty, as determined during the method validation study.

In case that the analyte content is below LOD or LOQ the result will be reported as below LOD or below LOQ respectively, and the concentration corresponding to the LOD / LOQ of the method will be provided.

If the calculated analyte content exceeds the upper limit of the working range, the sample shall be re-analysed with an adjusted, lower sample intake.

# Sample analysis by GC-MS

Before starting the sequence a solvent blank - toluene (4.9) - and the standard solution CS2 (see Table 5) have to be injected to verify the instrumental performances for this specific method.

The chromatogram of the solvent blank is applied to evaluate potential carry over.

## GC-MS operating conditions

### Injection conditions

Injetion type: splitless (2 min)

Injection volume: 1 μl

Injection speed: 5 μl/s

Pre injection delay: 1 s

Post injection delay: 1 s

Injector temperature: 250 °C

Purge flow: 50 ml/min

Purge time: 2 min

Total flow: 53.7 ml/min

Gas type: Helium (5.2)

### Oven conditions

Initial temperature: 70 °C

Initial time: 2 min

Ramp at 40 °C/min up to 180 °C, static time 0 min
Ramp at 10 °C/min up to 220 °C, static time 0 min
Ramp at 40 °C/min up to 280 °C, static time 10 min
(GC analysis time 20.25 min)

### Column conditions

Carrier gas flow: 1 ml/min (constant flow mode)

Gas type: Helium (5.2)

### Transfer line conditions

Temperature: 325 °C

### Mass spectrometer conditions

MS source temperature: 250 °C

MS Quadrupole temperature: 150 °C

Solvent delay: 5 min

Electron Ionisation Energy: 70 eV

### Mass spectrometer acquisition parameters and peak identification

The analytes are ionised by electron ionization (EI) at 70 eV. The target ions are recorded in Single Ion Monitoring (SIM) mode, and quantified by an isotope dilution method. Ionisation parameters and detection parameters are presented in Table 10 and Table 11 respectively.

Table 10. Ionisation parameters for Waters Quattro Micro GC

| Mode | EI+ |
| --- | --- |
| Electron energy | 70 eV |
| Trap current | 200 µA |
| Repeller | 4.8 V |
| Extraction lens | 14 V |
| Focus lens 1 | 35 V |
| Focus lens 3 | 42 V |
| Source temperature | 280°C |
| GC Interface temperature | 300°C |
| Multiplier voltage | 650 V |

Table 11. SIM detection parameters for Waters Quattro Micro GC.

| Time window (min) | recorded ion (m/z) | Dwell Time (s) | Compound |  |
| --- | --- | --- | --- | --- |
| 3.0-16.5 | 117 | 0.100 | Indole^†^ |  |
|  | 90 | 0.100 | Indole^‡^ |  |
|  | 123 | 0.100 | Indole-d7^†^ |  |
|  |  |  |  |  |
|  | 130 | 0.100 | Skatole^†^ |  |
|  | 103 | 0.100 | Skatole^‡^ |  |
|  | 132 | 0.100 | Skatole-d3^†^ |  |
|  |  |  |  |  |
|  | 151 | 0.100 | 5-Chloroindole^†^ |  |
|  |  |  |  |  |
| 16.6-29.0 | 272 | 0.200 | Androstenone^†^ |  |
|  | 257 | 0.200 | Androstenone^‡^ |  |
|  | 276 | 0.200 | Androstenone-d4^†^ |  |
| ^†^: quantifier SIM | | | | |
| ^‡^: qualifier SIM | | | | |
|  | | | | |
|  | | | | |

Figure 4 GC-MS (SIM) chromatogram of analysis of lard sample spiked with indole, skatole and androstenone at 100 µg/kg.

## Calibration curve

Calibration is performed by triplicate injections of 1 µL of the calibration solutions, randomized along the sequence. All solutions shall be equilibrated to room temperature prior to injection.

The calibration curve is obtained by plotting the peak area ratio of the quantifier ion of the target compounds to its corresponding labelled standard against the relative concentration of these compounds in the calibration solutions.

## Sample analysis

Analogue to chapter 8.2

# Calculation and reporting

Analogue to chapter 8.3

# Annex 1

In principle, the analysis can also be performed employing classical HPLC systems, whereas care has to be taken to guarantee sufficient chromatographic resolution and sensitivity.

HPLC conditions:

Column: Waters Symmetry C18 150 x 2.1 mm 3.5 μm

Injection volume: 5 μL

Column temperature: 35 °C

Flow rate: 0.3 mL/min

Gradient:

| Time | %LC eluent A (4.15) | Curve |
| --- | --- | --- |
| 0 | 50 | 6 |
| 1.5 | 50 | 6 |
| 10 | 10 | 6 |
| 13 | 4 | 6 |
| 13.1 | 50 | 6 |
| 18 | 50 | 6 |

Indicative retention times are presented in Table 12.

Table 12. Indicative analyte retention times.

| **Compound** | **Retention time (min)** |
| --- | --- |
| Indole | 3.31 |
| Indole-d7 | 3.31 |
| Skatole | 4.76 |
| Skatole-d3 | 4.76 |
| 5-Chloroindole | 5.61 |
| Androstenone | 11.97 |
| Androstenone-d4 | 11.97 |

For the MS method only the time windows need to be adjusted (Table 13) all other parameters are identical with those used for UHPLC.

Table 13. SRM detection parameters for HPLC-MS/MS analysis on Waters Quattro Premier instrument

| Time | SRM Transition | Dwell Time (s) | Cone Voltage | Collision Energy (eV) | Compound |
| --- | --- | --- | --- | --- | --- |
| 0.0-4.0 | 117.90 > 65.01 | 0.100 | 40.00 | 29.00 | Indole^‡^ |
|  | 117.90 > 90.97 | 0.100 | 40.00 | 20.00 | Indole^†^ |
|  | 123.97 > 95.88 | 0.100 | 40.00 | 20.00 | Indole-d7^†^ |
|  |  |  |  |  |  |
| 4.0-8.0 | 131.98 > 89.80 | 0.100 | 34.00 | 31.00 | Skatole^‡^ |
|  | 131.98 > 116.90 | 0.100 | 34.00 | 23.00 | Skatole^†^ |
|  | 134.98 > 116.90 | 0.100 | 34.00 | 23.00 | Skatole-d3^†^ |
|  | 151.95 > 116.95 | 0.100 | 30.00 | 18.00 | 5-Chloroindole^†^ |
|  |  |  |  |  |  |
| 8.0- 18.0 | 273.23 > 158.37 | 0.100 | 30.00 | 17.00 | Androstenone^‡^ |
|  | 273.23 > 255.12 | 0.100 | 30.00 | 14.00 | Androstenone^†^ |
|  | 277.23 > 259.12 | 0.100 | 30.00 | 14.00 | Androstenone-d4^†^ |
| ^†^: quantifier SRM transition | | | | | |
| ^‡^: qualifier SRM transition | | | | | |

LA-NA-26712-EN-N

1. e.g. Agilent 6890 GC with Waters Quattro Micro GC mass spectrometer [↑](#footnote-ref-1)
2. e.g. Waters Acquity UHPLC with Quattro Premier tandem mass spectrometer [↑](#footnote-ref-2)
