# Supplementary material for: Validation by collaborative trial of a method for the determination by GC–MS and LC–MS/MS of boar taint marker compounds in pork tissue
Source: Food Chem X. 2020 Mar 4;6:100083. doi: 10.1016/j.fochx.2020.100083 (PMC7082526; doi:10.1016/j.fochx.2020.100083)

**Supplementary Figure 1: Mandel's h plots for indole, skatole, and androstenone**

Thresholds for suspicious performance are set on the 5% significance level (yellow lines) and for outliers at the 1 % significance level (red lines). Columns indicating Mandel's h values exceeding the threshold values are coloured accordingly.


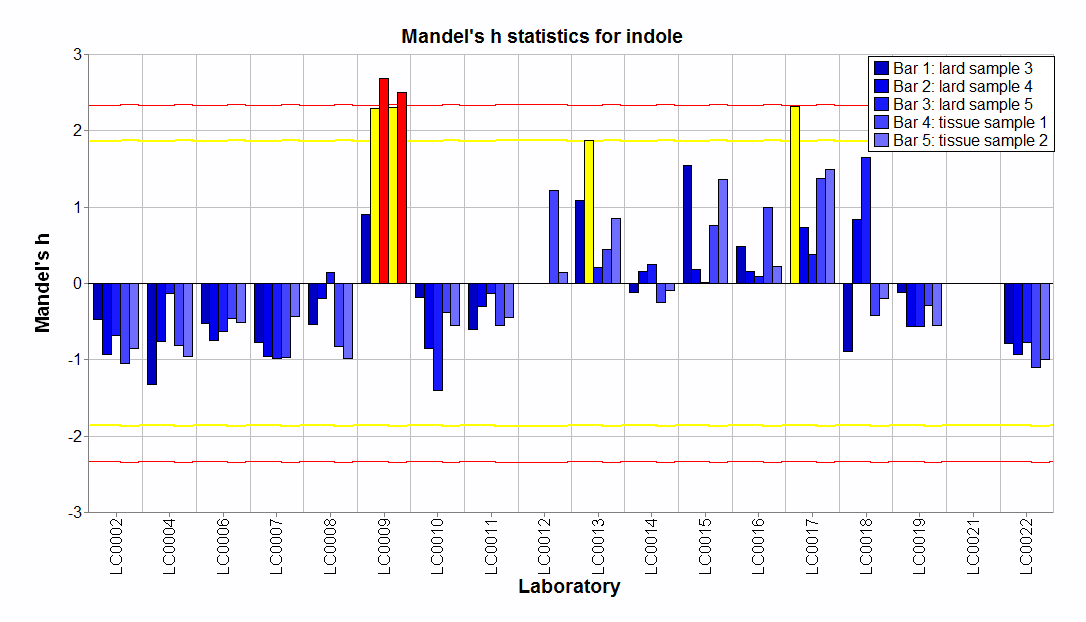


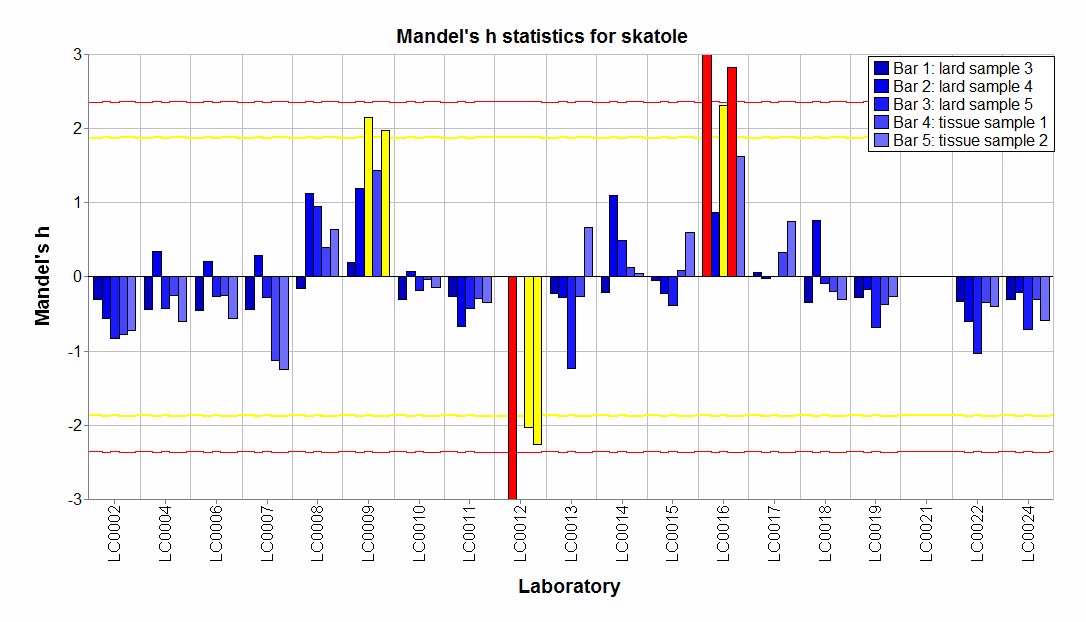


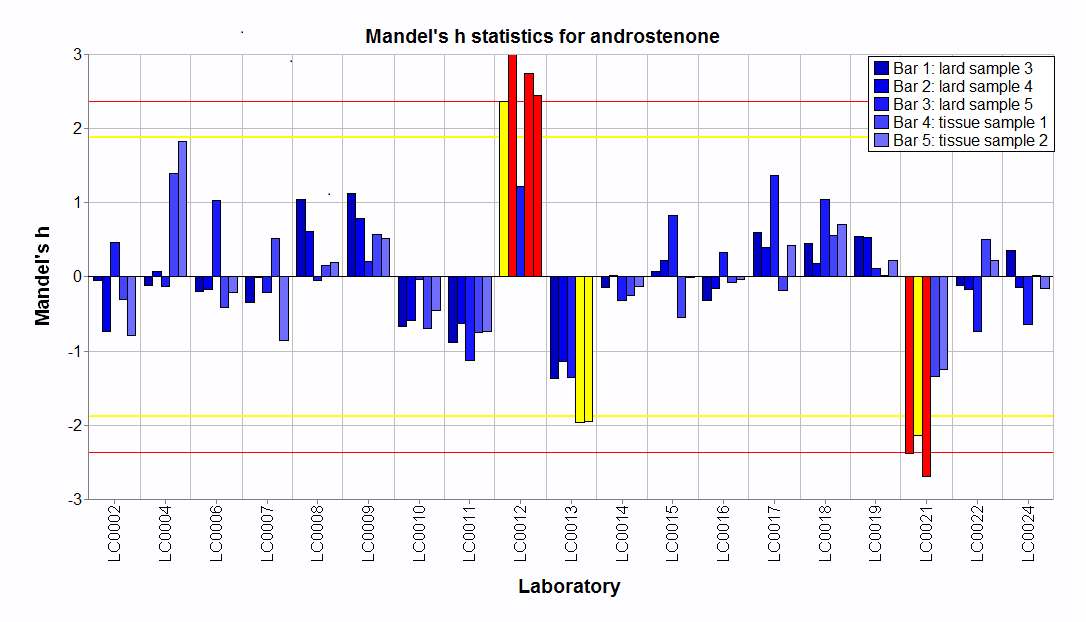

Supplement: Supplementary data 2 [file mmc2.docx]
